# 

**Published:** 1823-12-01

**Authors:** 


### Contents

OF

No. 15, Vol. IV. for DECEMBER 1823.

Art. I.

M. Tacheron's Anatomico-Pathological Researches in Practical Medicine - - - - - - - - - - - - - - 487

II.

Sketches in Bedlam; or Characteristic Traits of Insanity - - 511

III.

Transactions of the Associated Apothecaries and Surgeon-Apothecaries, Vol. I.

Art. 1. An Essay on the Education and Duties of the General Practitioner in Medicine and Surgery. By Thomas Alcock - - - - - - - - - - - - - - - - - 519

2. Dr. Johnson's Case of Chronic Carditis - - - - 548

IV.

Dr. Chapman's Elements of Therapeutics and Materia Medica (concluded from page 77) - - - - - - - - - - - 551

V.

On Thoracic Percussion and Abdominal Compression - - 569

VI.

Mr. Percivall's Elementary Lectures on the Veterinary Art 583

VII.

Dr. Paris and Mr. Fonblanque on Medical Jurisprudence (continued from page 426.) - - - - - - - - - 596

VIII.

(Retrospective.)

Mr. Charles Mansfield Clarke on those Diseases of Females attended by Discharges.

Part I. On Mucous Discharges - - - - - - - - - 635

IX.

Mr. Guthrie's Lectures on the Operative Surgery of the Eye (concluded from p. 448.) - - - - - - - - - - - 658

### X.

### Quarterly Periscope,

OR,

### *SPIRIT OF THE PUBLIC JOURNALS.*

1. Dr. Andral's Researches on the Pathological Anatomy of the Digestive Canal - - - - - - - - - - - - - 673
2. Dr. Price on the Management of Leeches - - - - - 697
3. Dr. Scoulton on Meningine - - - - - - - - - - - 698
4. Mr. Wansbrough's Case of Poisoned Wound - - - 698
5. Mr. Ward on Abortion - - - - - - - - - - - - 699
6. Dr. Lavagna on Amenorrhœa - - - - - - - - - - 700
7. Dr. Carter on Epilepsy - - - - - - - - - - - - 700
8. Dr. Crawford on Tic Douloureux - - - - - - - - 700
9. Dr. Pommer on Antimoniated Ointment - - - - - 701
10. Destruction of a Portion of Spinal Marrow - - - - 701
11. On Extirpation of the Thyroid Gland - - - - - - - 702
12. M. Fleurens on the Nervous System - - - - - - - 703
13. Enlargement of the Heart - - - - - - - - - - - 706

### XI.

FRACTURES OF THE CERVIX FEMORIS.

1. Practical Observations in Surgery. By Henry Earle, Esq. F.R.S.
2. Observations on Fractures of the Neck of the Thigh-Bone. By Sir Astley Cooper, Bart. &c. - - - - - - - 707

### XII.

Bibliographical Record - - - - - - - - - - - - - - 729

### XIII.

Biography—Dr. Baillie - - - - - - - - - - - - - 733

### XIV.

MISCELLANEOUS INTELLIGENCE.

Dr. Merriman to Dr. Johnson - - - - - - - - - - 738
Ulceration of the Cæcum - - - - - - - - - - - - - - 739
German Translation of Dr. W. Philip's Work on Indigestion 741
Transactions of Associated Apothecaries - - - - - - - 741
Clapham Retreat - - - - - - - - - - - - - - - - - 741
Dr. Smith's Lectures on Medical Jurisprudence - - - - 741
Dr. Harrison's Letter to the Editor - - - - - - - - - 742
Injecting Machine for the Stomach and Bowels - - - - - 742

